# The therapeutic potential for targeting CSE/H_2_S signaling in macrophages against *Escherichia coli* infection

**DOI:** 10.1186/s13567-023-01203-8

**Published:** 2023-08-29

**Authors:** Shaodong Fu, Zhenglei Wang, Xiangan Han, Yuanyuan Xu, Jinfeng Miao

**Affiliations:** 1 MOE Joint International Research Laboratory of Animal Health and Food Safety, Key Laboratory of Animal Physiology and Biochemistry, College of Veterinary Medicine, Nanjing Agricultural University, Nanjing, 210095 China; 2 Shanghai Veterinary Research Institute, Chinese Academy of Agricultural Sciences, Shanghai, 200241 China

**Keywords:** Cystathionine γ-lyase, macrophages, *Escherichia coli*, autophagy, inflammation

## Abstract

Macrophages play a pivotal role in the inflammatory response to the zoonotic pathogen *E. coli*, responsible for causing enteric infections. While considerable research has been conducted to comprehend the pathogenesis of this disease, scant attention devoted to host-derived H_2_S. Herein, we reported that *E. coli* infection enhanced the expression of CSE in macrophages, accompanied by a significantly increased inflammatory response. This process may be mediated by the involvement of excessive autophagy. Inhibition of AMPK or autophagy with pharmacological inhibitors could alleviate the inflammation. Additionally, cell model showed that the mRNA expression of classic inflammatory factors (Il-1β, Il-6), macrophage polarization markers (iNOS, Arg1) and ROS production was significantly down-regulated after employing CSE specific inhibitor PAG. And PAG is capable of inhibiting excessive autophagy through the LKB1-AMPK-ULK1 axis. Interestingly, exogenous H_2_S could suppress inflammation response. Our study emphasizes the importance of CSE in regulating the macrophage-mediated response to *E. coli*. Increased CSE in macrophages leads to excessive inflammation, which should be considered a new target for drug development to treat intestinal infection.

**Supplementary Information:**

The online version contains supplementary material available at 10.1186/s13567-023-01203-8.

## Introduction

*Escherichia coli *(*E. coli*) are present in both humans and farm animals, is a leading cause of acute diarrhea worldwide [1]. Emerging drug-resistant bacteria and the cliff-like reduction of new antibacterial drugs sharpened the difficulty in the treatment of *E. coli* infections, giving rise to significant morbidity and mortality [2]. In developing regions, diarrheagenic *E. coli* can cause up to 40% of diarrhea in children under five [3]. Apart from being an anaerobic bioreactor programmed with a huge population of bacteria, the intestinal lumen ultimately serves as a natural reservoir for antibiotic resistance genes (ARGs), making it easy for plasmid-mediated horizontal transfer of ARGs occurs during antibiotic therapy [4]. In China, the number of ARGs per isolate animal-derived *E. coli* samples collected from the 1970s to 2019 has doubled and poses a significant threat to public health [5]. Therefore, it is urgent to develop safer and more effective strategies to combat *E. coli* infections.

Macrophages residing in intestinal tissues are crucial for maintaining intestinal homeostasis and preventing disease development [6]. These cells play a vital role in inflammation resolution and can be classified into two subtypes based on microenvironmental cues: the classically activated M1 macrophages and the alternatively activated M2 macrophages. While M1 macrophages secrete proinflammatory cytokines like interleukin-1 (IL-1) and tumor necrosis factor-alpha (TNF-α), exacerbating inflammation, M2 macrophages produce anti-inflammatory cytokines like interleukin-10 (IL-10), promoting tissue regeneration and alleviating inflammation [7]. However, excessive activation of M2 macrophages can impair intestinal protective function and worsen intestinal damage [8]. Therefore, it is crucial to examine how the chemotaxis, proliferation, differentiation, and activation of macrophages are regulated during intestinal infection. Such understanding can provide insights into the mechanisms and new treatment options for intestinal inflammatory diseases.

Hydrogen sulfide (hereafter referred to as H_2_S) the latest identified endogenous gaseous mediator, was once considered a foul-smelling and hazardous gas found in the environment [9]. Recently, H_2_S has gained increasing attention as an important player in modulating a wide range of physiological and pathological conditions [10, 11]. In mammals, similar to NO and CO, three enzymes including cystathionine γ-lyase (CSE), cystathionine β-synthase (CBS) and the 3-mercaptopyruvate sulphur transferase (3-MPST) are responsible for H_2_S generation. Specifically, CSE and CBS promote de-sulphydration of l-cysteine to generate H_2_S, while 3-MPST induces H_2_S production by controlling the enzymatic activity of cysteine aminotransferase [12]. In fact, the effects of H_2_S are often bell-shaped: beneficial at normal physiological concentrations but toxic at high doses due to its inhibition of mitochondrial complex IV. Aberrations of CBS and CSE can lead to different outcomes, as H_2_S has both anti-inflammatory and pro-inflammatory effects [13]. Autophagy is a fundamental process involving the delivery of cellular material to lysosomes for degradation and recycling that contributes to cellular and tissue homeostasis, physiology, and development. However, abnormalities in autophagy may contribute to many different pathophysiological conditions [14]. There is an increasing amount of evidence suggesting that both endogenous and exogenous H_2_S could exhibit two evidently opposite effects on autophagy [15]. Therefore, it is urgent and essential to elucidate the mechanism of action of H_2_S in the autophagy process.

In this study, we have observed that an *E. coli* infection in the intestine can trigger an immune response that results in enhanced macrophage activity and recruitment to the site of infection in vivo. Additionally, in vitro experiments have shown that an increase in CSE expression of macrophages occurs in response to *E. coli*, accompanied by a significant rise in inflammation that may be associated with excessive autophagy. Both inhibiting CSE expression or autophagy can reduce inflammation. These findings shed light on the molecular mechanisms involved in antimicrobial defense and regulation of host sulfur metabolism, providing a potential strategy to address the growing issue of enteric infectious diseases.

## Materials and methods

### Bacterial strains, cell culture, and treatment

*Escherichia coli *(strain ATCC 25922) was inoculated into Luria–Bertani (LB) culture and incubated at 37 °C in an orbital shaker to log-phase growth OD_600_ = 0.4–0.6. RAW264.7 cells were incubated in Dulbecco’s modified Eagle’s medium (DMEM) with 10% fetal bovine serum (Evergreen, Huzhou, China) and plated at 80% confluence in a 6-well cell plates, the monolayers were treated with or without 500 µM PAG (inhibitor of CSE: Aladdin, Shanghai, China) for 4 h, 10 µM Choroquine (inhibitor of autophagy, TargetMol, Boston, USA) and Compound C (inhibitor of AMPK, Selleck Chemicals, Houston, USA) for 2 h, 200 µM GYY4137 (Sigma-Aldrich, MO, USA) for 24 h, all at 37 °C. The treated cells were infected with *E. coli* at a multiplicity of infection (MOI) of 10 at 37 ℃ according to different test conditions. The supernatant and cells were collected separately and stored at −80 ℃ until use. Other reagents and materials are listed in Additional file 1.

### Mice, and establishment of infection model

All animal experiments were carried out in accordance with the animal welfare standards and complied with the guidelines of the Animal Welfare Council of China and was approved by the Ethical Committee for Animal Experiments of Nanjing Agricultural University (NJAU. No20220509099). Mice were randomly divided into two groups (control group and infected group). One hundred microliters of 5% NaHCO_3_ was inoculated by oral gavage to neutralize gastric acidity, and 30 min after this treatment, 2 × 10^10^ CFU in 100 μL *E. coli* ATCC 25922 was inoculated by oral gavage, and mice in control group were gavaged with equivalent volume of normal saline solution. Following the 24 h time point, mice were sacrificed, corresponding samples were collected and stored at −80 °C until use.

### RNA extraction and quantitative real-time polymerase chain reaction (qPCR)

Total RNA was extracted by TRIzol reagent (AG, Changsha, China). The corresponding cDNA was obtained using reverse transcriptase (AG, Changsha, China). An aliquot of cDNA was mixed with 5 µL TB Green PCR Master Mix (AG, Changsha, China) and 0.2 µL of each specific forward and reverse primer. All mixed systems were analyzed in a Roche LightCycler 96. *β-actin* was set as reference gene, and fold changes were calculated as 2^−ΔΔCt^. All primer sequences (Additional file 2) were synthesized by Tsingke Biological Company (Nanjing, China).

### Total protein extraction and Western blotting

Cells were washed three times on ice-cold phosphate buffered saline and lysed by incubation in RIPA Lysis buffer containing protease inhibitor phenylmethanesulfonyl fluoride (Beyotime, Nantong, China). After grinding, supernatants were collected by centrifugation (5000 × *g* for 10 min at 4 °C). Protein concentration was determined by bicinchoninic acid assay (BCA kit, Beyotime, Nantong, China). Extracts with equal amounts of protein were solubilized in SDS sample buffer, separated by SDS–PAGE, transferred to polyvinylidene difluoride membranes (Millipore), blocked with 5% nonfat milk diluted in Tris-buffered saline with Tween‐20 (TBST) for 2 h at room temperature, and hybridized with primary antibody at 4 ℃ overnight. Before and after incubation with the secondary antibodies for 2 h at room temperature, the membranes were washed three times for 15 min with TBST. Secondary antibody was HRP‐linked anti‐rabbit IgG (CST; 1:10 000). Signals were detected by an ECL Western Blot Analysis System (Tanon, Shanghai, China). Bands were quantified with ImageJ 1.8 software (NIH, Bethesda, MD, USA).

### Immunohistochemical staining

The immunohistochemistry procedures were carried out following previously described with minor modifications [16]. First, sections were sliced from paraffin-embedded specimens, deparaffinized in xylene and hydrated in a graded series of ethanol, placed in 0.01 mol/L citrate buffer (pH 6.0), and heated in a microwave oven over medium–high heat for 10 min. Then, the slices were incubated with 3% H_2_O_2_-methanol solution at room temperature for 10 min. Following primary and HRP-conjugated secondary antibody incubation, diaminobenzidine was used as a chromogen and hematoxylin for counterstaining. Finally, the slices were observed under a microscope and photographed.

### BODIPY staining and microscopy

Cells in twelve-well plate were washed three times with PBS and fixed with 4% formaldehyde for 15 min at room temperature. Then were again washed 3 times with PBS after incubation with 2 µM BODIPY in the dark for 15 min at 37 ℃. Next, they were then incubated with 20 µM DAPI in the dark for 10 min at room temperature and washed 3 times in PBS. Finally, the plate was photographed inverted fluorescence microscope (EVOS FL Auto 2, Invitrogen).

### Assessment of biochemical parameters

The protein samples from cells were extracted using cold lysis bufer and assayed using a BCA Kit. The levels of total cholesterol (TC), triglycerides (TGs), lactate dehydrogenase (LDH) and endogenous H_2_S, in cell lysates were measured using commercial kits according to the manufacturer’s instructions (Jiancheng, Nanjing, China).

### Statistical analyses

Statistical analyses were performed using GraphPad Prism 8 software. Differences were evaluated by unpaired t test or one-way ANOVA followed by Tukeys post hoc tests. All data are represented as the means ± standard error of the mean (SEM). The significance level was set as *P* < 0.05.

## Results

### *E. coli* infection alters CSE levels in macrophages

**Figure 1:**
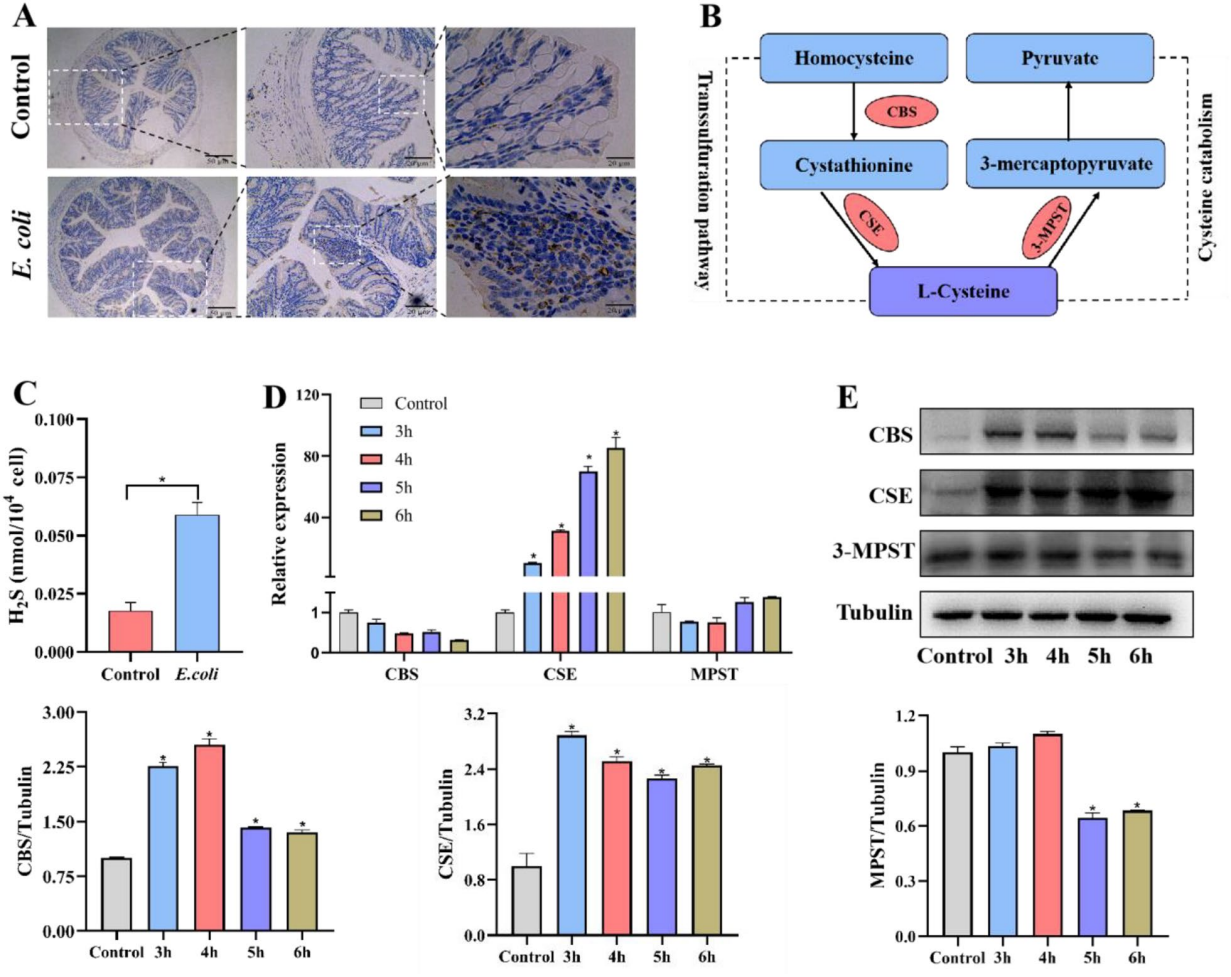
***E. coli ***infection lead to an elevation of CSE in macrophages. **A** Positive staining for macrophage marker CD68 during ***E. coli*** infection. **B** Pathways of transsulfuration pathway in mammalian. **C** Detection of endogenous H_2_S in RAW264.7 cells during ***E. coli*** challenge. Relative mRNA expression **D** and protein expression levels (**E**) related to H_2_S synthesis in RAW264.7 cells during ***E. coli*** invasion. Data are presented as the means ± SEMs (*n* = 3). *(*P* < 0.05) = significantly different.

When a pathogen invades an organism, macrophages are recruited through migration and release cytokines to induce an inflammatory response. The infiltration of macrophages was identified by immunohistochemical staining for the macrophage marker CD68 (Figure 1A). In view of the significant increase of macrophage infiltration after *E. coli* infection, we were becoming inquisitive about the contributor to this. The sulfur metabolism pathway plays an important role in organisms, especially transsulfuration pathway (Figure 1B). Infection of RAW 264.7 macrophages with *E. coli* resulted in a threefold increase in the level of H_2_S after 6 h infection (*P* < 0.05), This suggests that *E. coli* infection can cause an increase in H_2_S levels, which can be detected. Consequently, it is necessary to examine the key enzymes involved in H_2_S synthesis during dynamic infection process (Figure 1C). First, qPCR assay demonstrated CSE expression fold-change increased significantly at the transcriptional level in a time-dependent manner upon *E. coli* stimulation, comparing to other two enzymes (*P* < 0.05) (Figure 1D). This observation was further confirmed by Western blot analysis (*P* < 0.05) (Figure 1E). And we noticed that CBS levels increased significantly within 3–4 h of infection and both CBS and 3-MPST protein decreased within 5–6 h. Following a thorough analysis, we chose to conduct our subsequent experiment at 5 h of infection time. At this point, H_2_S generation is likely to be mainly driven by CSE.

### CSE exacerbates the pro-inflammatory response of macrophages exposure to* E. coli*

**Figure 2:**
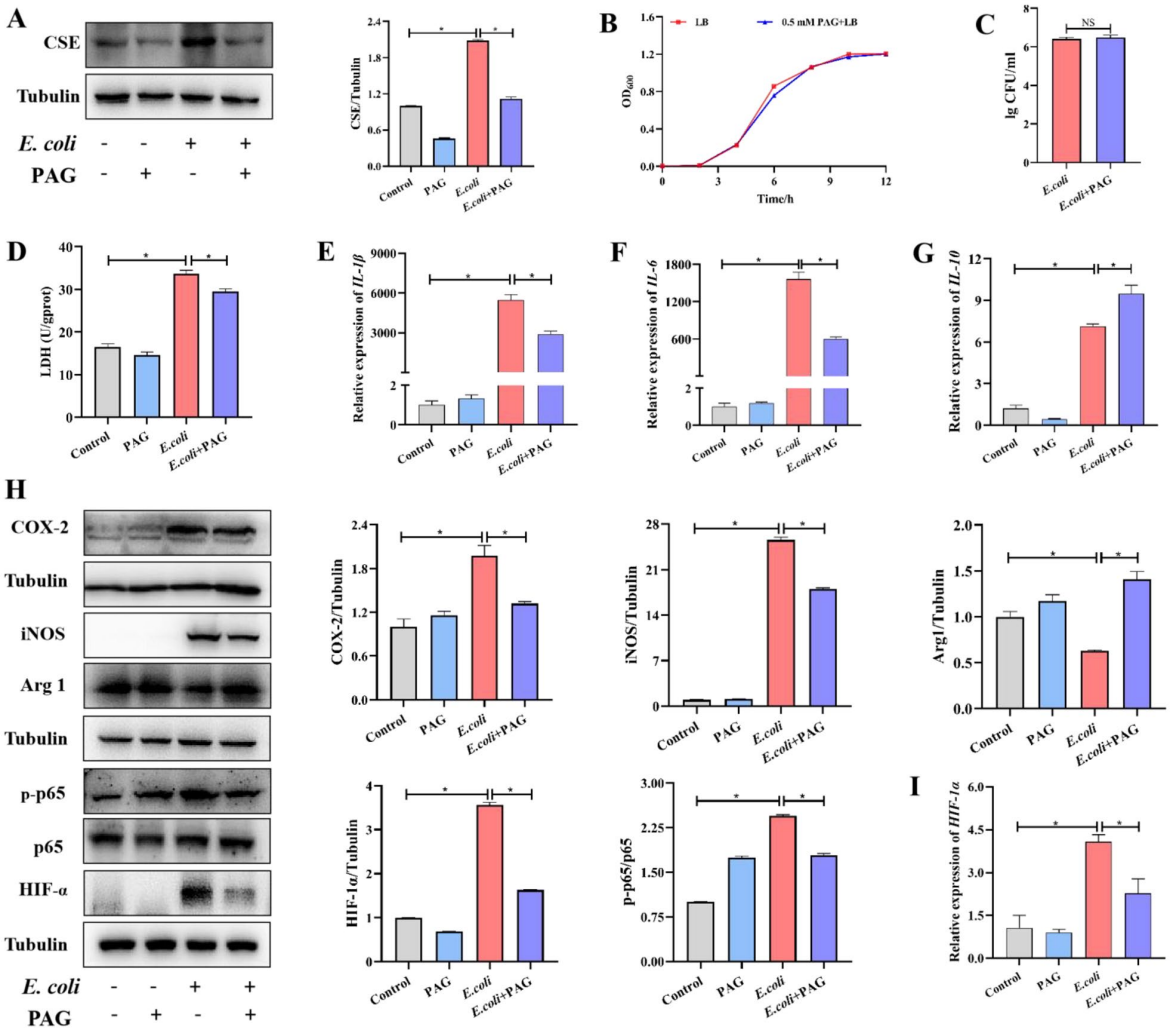
CSE amplified the inflammatory response induced by ***E. coli***. **A** Effect of CSE inhibition by PAG in RAW264.7 cells during ***E. coli*** infection. **B** Bacterial growth curves supplemented with PAG. **C** The impact of macrophage phagocytosis with PAG supplementation. Assessment of lactate dehydrogenase (LDH) activity (**D**) in the supernatants of RAW264.7 cells primed with PAG during ***E. coli*** infection. mRNA expression levels of pro-inflammatory IL-1β (**E**), IL-6 (**F**) and anti-inflammatory factors IL-10 (**G**) in RAW264.7 cells during ***E. coli*** invasion with or without PAG treatment. **H** Inflammation related protein expression during ***E. coli*** challenge with or without PAG treatment. Data are presented as the means ± SEMs (*n* = 3). *(*P* < 0.05) = significantly different.

To investigate the role of CSE in the pathogenesis of *E. coli* in the macrophage, a specific CSE inhibitor PAG was used to detect the effects. The effect of inhibitory effect was confirmed by performing Western blot analysis (Figure 2A), and take no effect on the growth of *E. coli* and the phagocytic capacity of macrophages (Figures 2B, C). As a rule, lactate dehydrogenase (LDH) release into the culture medium is an indicator for cell damage. And we found LDH release (Figure 2D) markedly decreased after employing PAG (*P* < 0.05). By detecting the mRNA levels of multiple cytokines in macrophages, we found that *E. coli* infection caused a sharp increase in the expression of Il-1β (Figure 2E), TNF-α (Figure 2F). Il-1β increased nearly 7000 times (*P* < 0.05). PAG pretreatment significantly decreased mRNA levels (*P* < 0.05), however, the mRNA levels of anti-inflammatory factors IL-10 (Figure 2G) show an opposite trend (*P* < 0.05). As shown in Figure 2I *E. coli* challenged significantly increased the pro-inflammatory factors of p65 phosphorylation levels and COX-2 protein (catalyzes the formation of prostaglandin) expression level, while PAG treatment significantly inhibited the above changes (*P* < 0.05). Meanwhile, the protein levels of iNOS and were significantly increased after infection and PAG reduced their expression levels (*P* < 0.05). Conversely, the protein level of Arg1 showed the opposite trend. These results were confirmed by the detection of HIF-1α both in transcript (Figure 2I) and protein level (*P* < 0.05). Conversely, these data demonstrated that up-regulated CSE could exacerbate inflammatory damage and affect the metabolism of macrophages induced by *E. coli* infection.

### *E. coli* infected macrophages reactive oxygen species production and polarization were related to CSE

**Figure 3:**
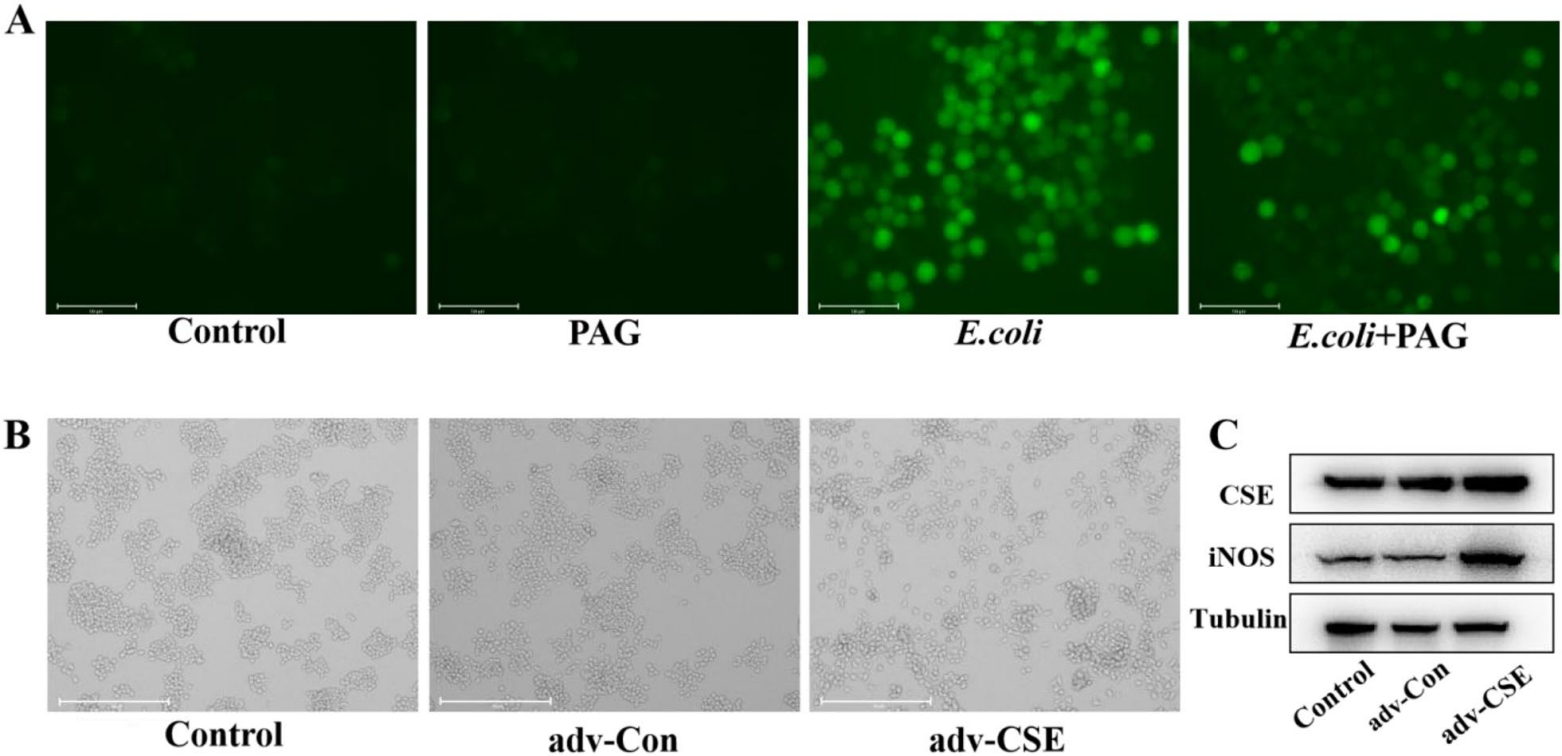
CSE promoted macrophages reactive oxygen species generation and polarization induced by ***E. coli***. **A** Reactive oxygen species were evaluated using DCFH-DA probes in RAW264.7 cells during ***E. coli*** invasion with or without PAG treatment. **B** RAW264.7 cells were transfected CSE overexpression plasmids and control cells were transfected with empty vector. **C** The overexpression of CSE was validated by Western blot analysis and the polarization marker iNOS detection.

To test the hypothesis that the release of reactive oxygen species (ROS) resulting from the aforementioned inflammatory condition, we examined the role of CSE in cellular ROS production in *E. coli-*infected macrophages, and found PAG intervention can effectively reduced the ROS level result from *E. coli* infection (Figure 3A). Furthermore, overexpression of CSE by plasmid transfection in RAW 264.7 cells was investigated, after gene transfection, we observed a significant alteration in cell morphology, with cells no longer clustered and producing antennae (Figure 3B). As iNOS expression is an important marker for M1 macrophage differentiation, the protein level of iNOS was increased after CSE overexpression (*P* < 0.05) (Figure 3C). Taken together, our data indicated that CSE engaged in the generation of ROS and polarizing macrophages.

### Upregulated CSE may involved in lipid metabolism upon* E. coli* infection

**Figure 4:**
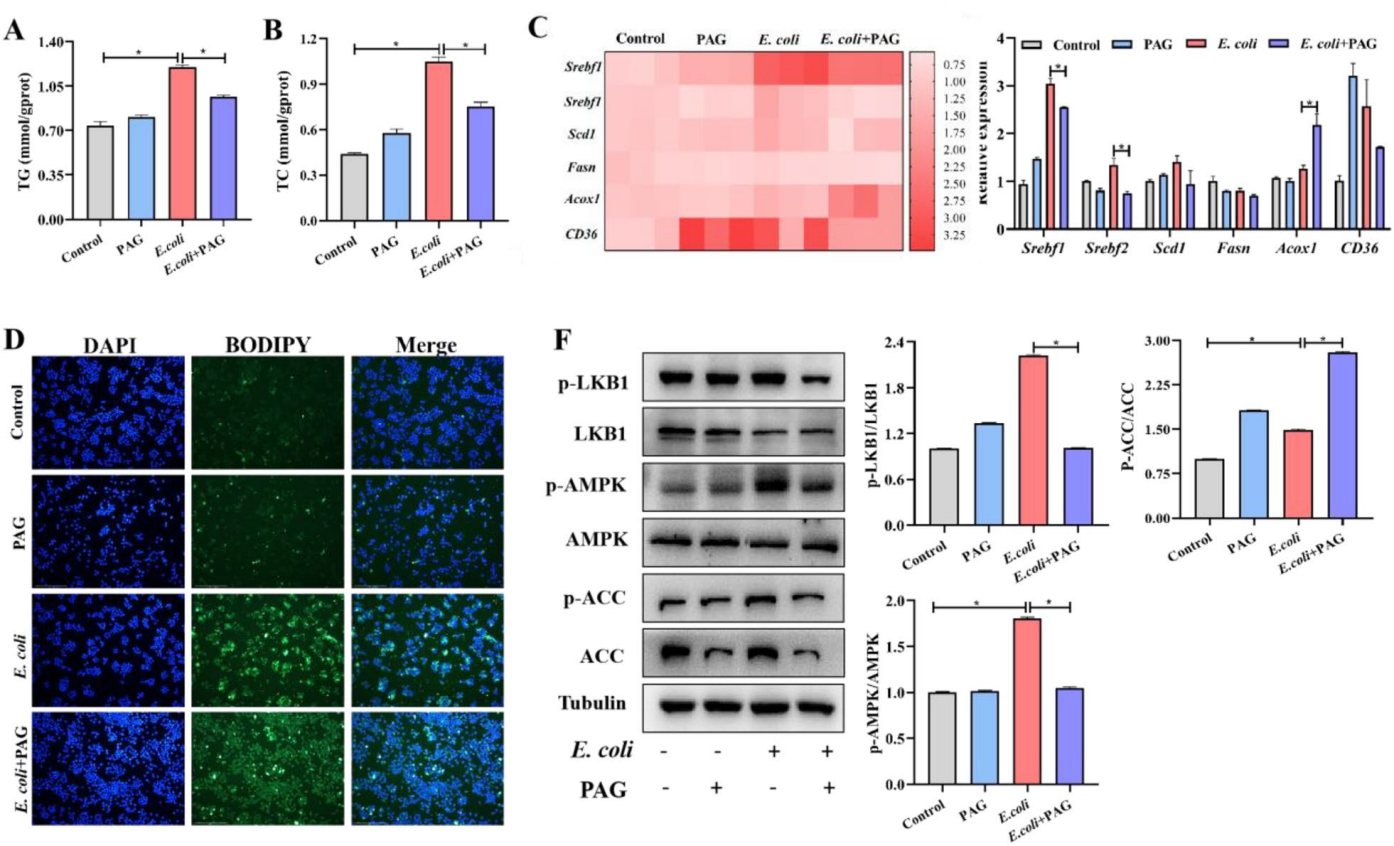
Inhibiting CSE can reduce macrophage lipid droplet accumulation against ***E. coli*** infection. **A**, **B** TG and TC contents in RAW264.7 cells during ***E. coli*** invasion. **C** Relative mRNA expression levels related to lipid synthesis in RAW264.7 cells during ***E. coli*** challenge. Heatmap (on the left) and statistical chart (on the right). **D** BODIPY 493/503 staining for LD visualization (green) increased in ***E. coli*** infection. **F** Expression of lipid metabolism related proteins in ***E. coli*** infection. Data are presented as the means ± SEMs (*n* = 3). *(*P* < 0.05) = significantly different.

Mounting studies have revealed that lipid droplets play a significant role in regulating lipid homeostasis and participating in inflammatory responses [17]. Additionally, CSE has been closely associated with lipid metabolism. Herein, we found that PAG pretreatment can dramatically lower the content of triglycerides (TGs) (Figure 4A) and total cholesterol (TC) (Figure 4B), which were significantly increased when encounter *E. coli* infection (*P* < 0.05). The aforementioned results were further confirmed by BODIPY staining analysis (Figure 4C). To validate these findings, we examined the mRNA levels of lipid metabolism related genes by qRT-PCR, *E. coli* stimulation increased expressions of *Srebf1*, *Srebf2*, while PAG suppressed the increased (*P* < 0.05), the variation tendency of *Acox1* was opposite to that (*P* < 0.05) and there were no significant changes in *Scd1* and *Fasn* genes (Figure 4D). Then we analyzed LKB1/AMPK/ACC signaling, which is a classical pathway involved in lipid metabolism regulation. Then results showed that *E. coli* infection could increase phosphorylation of LKB1 and AMPK, inhibition of CSE by PAG lead to the level of phosphorylation decrease (*P* < 0.05). Interestingly, phosphorylation of ACC continue to increase, which was different from our expectations (Figure 4E). Moreover, it can be seen that the use of PAG alone can also result in an increase in the phosphorylation of ACC, indicating PAG could potentially have a direct regulatory effect on ACC. Therefore, these findings indicate that CSE plays a role in regulating the LKB1/AMPK pathway related to lipid metabolism in response to *E. coli* infection, while the impact of PAG on ACC phosphorylation could be more significant than the influence of increased AMPK phosphorylation.

### CSE is responsible for inducing autophagy during an *E. coli* challenge

**Figure 5:**
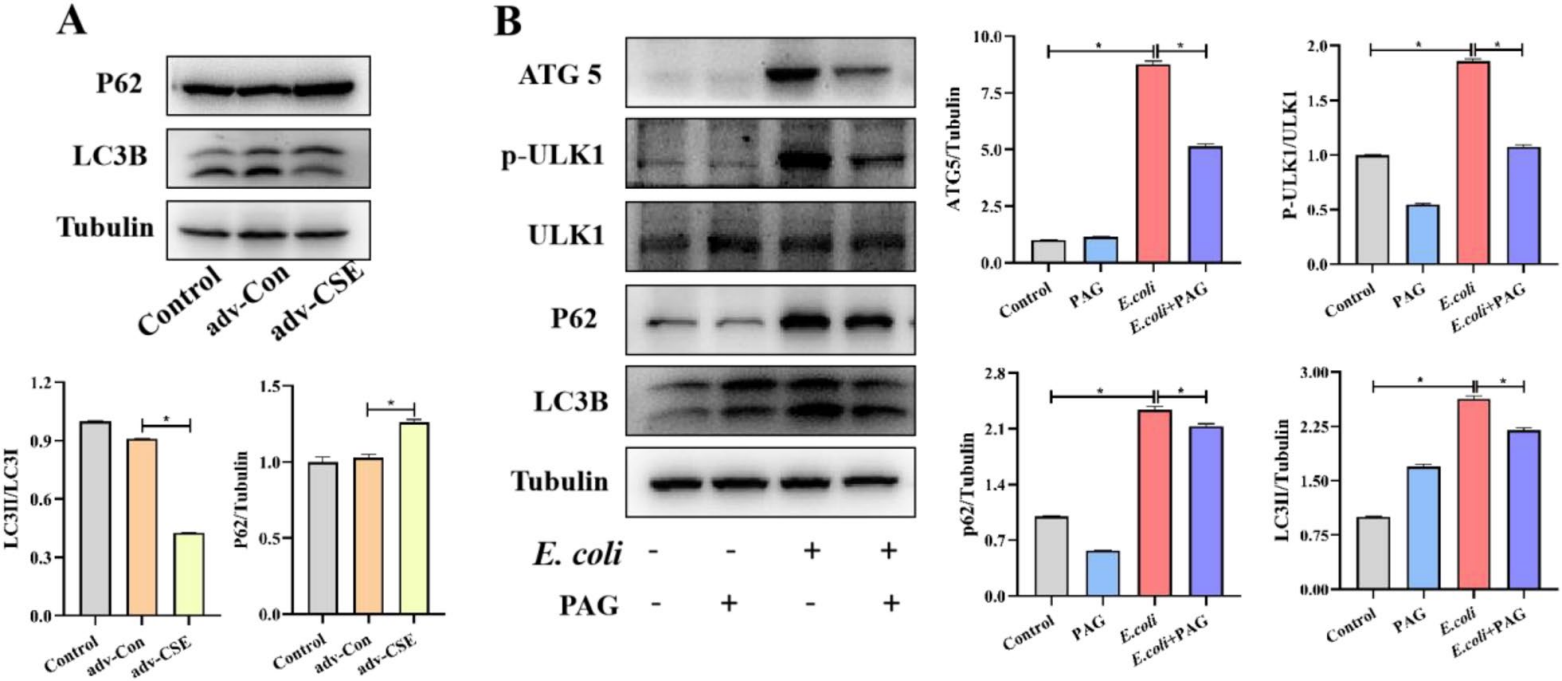
CSE induced autophagy during ***E. coli*** challenge. **A** RAW264.7 cells were transfected with CSE overexpression plasmids, while control cells were transfected with an empty vector. Immunoblots were performed to detect changes in autophagy-related proteins. **B** The signalling pathways connected to autophagy. during ***E. coli*** challenge with or without PAG treatment. Data are presented as the means ± SEMs (*n* = 3). *(*P* < 0.05) = significantly different.

Given that AMPK is the main sensor and regulator of cell metabolism, we try to explore how LKB1-AMPK function. We focused on its downstream molecule ULK1, the mammalian homolog of yeast Atg1, for autophagosome formation. In the absence of *E. coli* infection, CSE overexpression resulted in P62 protein increased, accompanying LC3 protein decreased (*P* < 0.05) (Figure 5A), which suggested a possible relevance of CSE with the autophagic pathway. For the purpose of further corroborating the above conclusion, we utilized PAG to reverse the observed impact of overexpressed CSE. Upon doing so, we observed that PAG was able to effectively reverse the influence of LC3 and p62 proteins resulting from CSE overexpression (Additional file 3). When cells were exposed to *E. coli*, we found the autophagy proteins ATG5, P62 and ULK1 phosphorylation increased markedly, however PAG can reverse such changes (*P* < 0.05). Instead, LC3 protein raised significantly, and a significant decreasing trend was observed after PAG pretreatment (*P* < 0.05) (Figure 5B). Overall, results above confirmed that *E. coli* infection activated AMPK-ULK1 and consequently promotes autophagy, while this phenomenon could be reversed by inhibiting CSE.

### Inhibiting excessive autophagy attenuated inflammation upon* E. coli* infection

**Figure 6:**
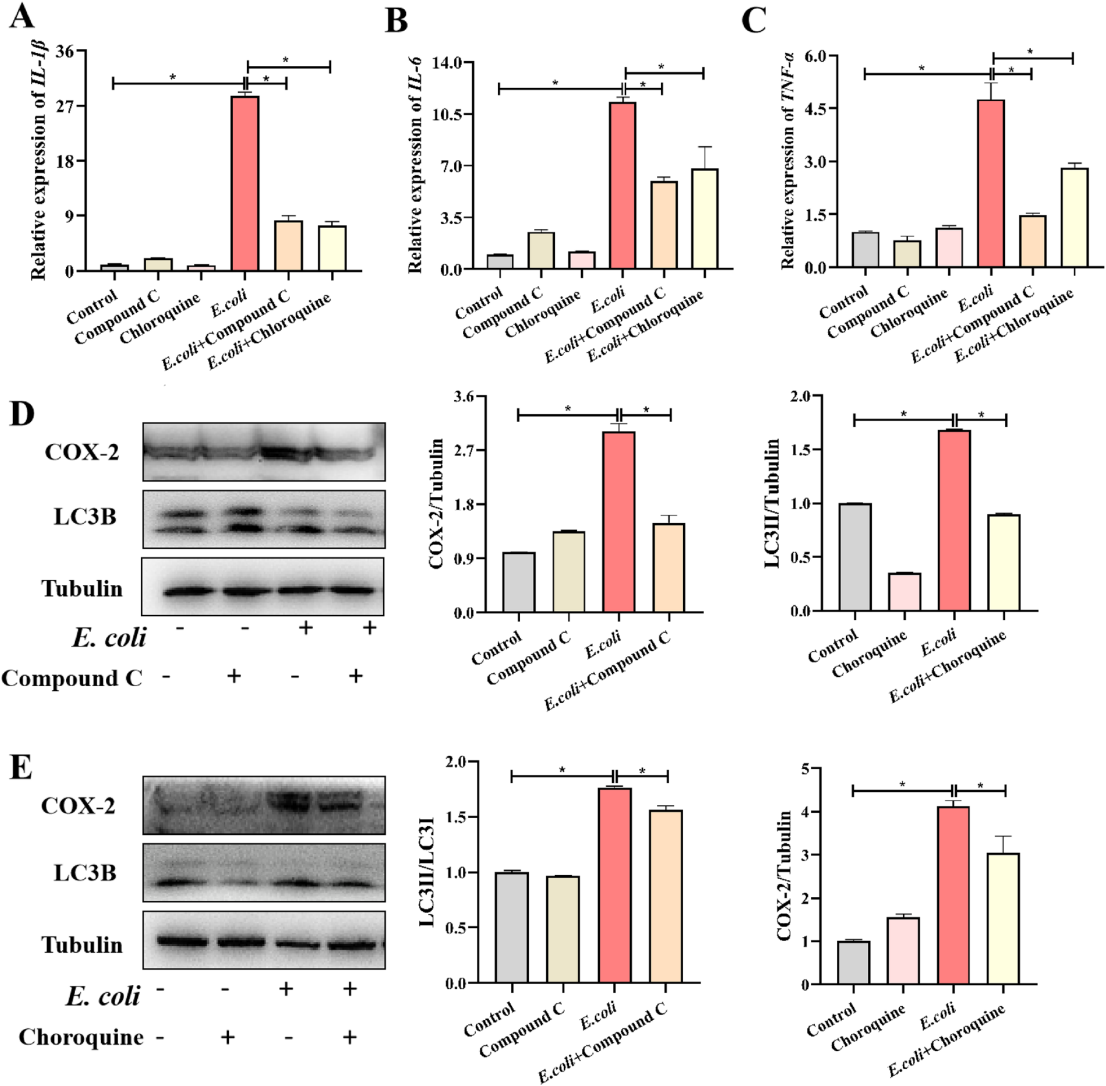
Inhibiting excessive autophagy resulted in the attenuated inflammation upon ***E. coli*** infection. Relative mRNA expression levels of pro-inflammatory IL-1β (**A**), IL-6 (**B**) and TNF-α (**C**) in RAW264.7 cells during ***E. coli*** invasion with or without compound C and chloroquine. **D** Immunoblots were performed to detect changes in autophagy-related protein (LC3B) and inflammatory-related proteins (COX-2), after pretreat compound C (**D**) and chloroquine (**E**). Data are presented as the means ± SEMs (*n* = 3). *(*P* < 0.05) = significantly different.

Since we observed that CSE affected autophagy in *E. coli*-infected macrophages, we asked whether autophagy could regulate inflammation. Hence, we used the AMPK inhibitor compound C and autophagy inhibitor chloroquine to treat *E. coli*-infected macrophages, and we found significant reduction of proinflammatory factors in mRNA expression levels after both compound C and chloroquine pretreatment, such as IL-1β (Figure 6A), IL-6 (Figure 6B), and TNF-α (*P* < 0.05) (Figure 6C). We then investigated COX-2 protein levels and LC3 protein levels using Western blotting. Like in the previous results, *E. coli* infection caused LC3 and COX-2 protein level increased. After employing two inhibitors, both LC3 and COX-2 protein level decreased (*P* < 0.05) (Figure 6D, E). Collectively, we have elucidated that inhibiting CSE attenuated excessive inflammation in an autophagic machinery-dependent manner.

### H_2_S donor GYY4137 displayed stronger protection against ***E. coli*** infection

**Figure 7:**
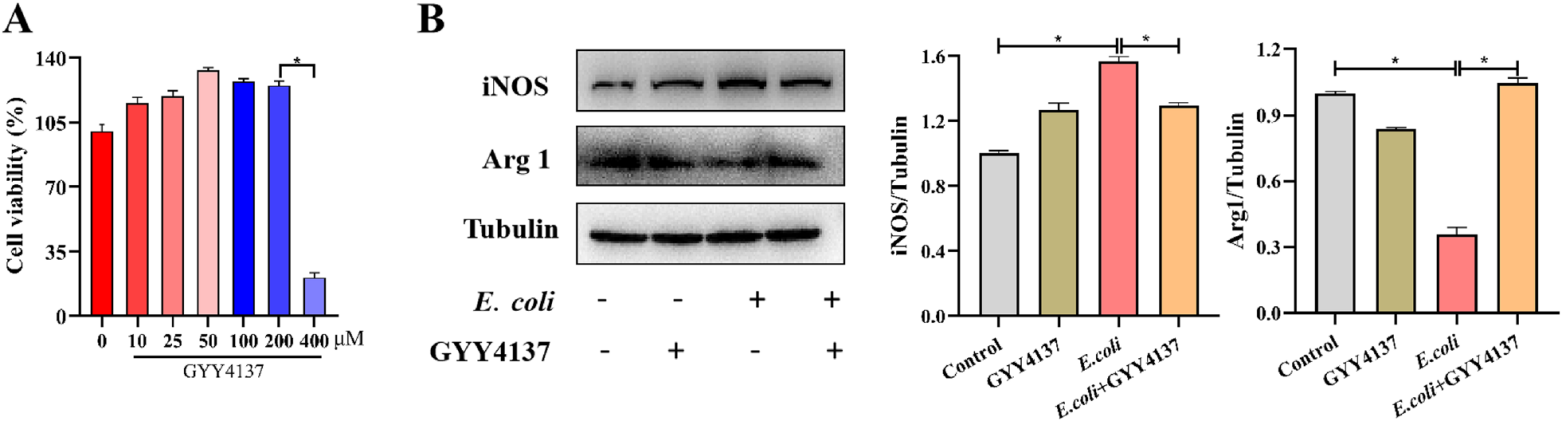
GYY4137 effectively protected against ***E. coli*** infection. A CCK8 assay for cellular viability. **B** Inflammation-related protein expression during ***E. coli*** challenge with or without GYY4137 treatment. Data are presented as the means ± SEMs (*n* = 3). *(*P* < 0.05) = significantly different.

Next, we investigated the role of exogenous H_2_S play in this process. Firstly, it was observed that GYY4137 was not cytotoxic to RAW 264.7 cells at concentrations under 200 μM (*P* < 0.05) (Figure 7A). We also examined the expression of iNOS and Arg1 protein using Western blot analysis and discovered that pretreatment with GYY4137 was able to effectively decrease the levels of iNOS protein whilst increasing Arg1 protein, thus partially reversing the changes caused by *E. coli* infection (*P* < 0.05) (Figure 7B). Overall, the role of exogenous H_2_S did not align with that of endogenous H_2_S in macrophages during *E. coli* infection.

## Discussion

*E. coli* is a significant contributor to infections globally, particularly diarrheal diseases, which result in considerable economic losses. The intestine houses the highest concentration of immune cells in the body, of which macrophages are predominant in the lamina propria [18]. To investigate the pathogenesis of enteric infection, we first developed infection models in mice through the oral administration of *E. coli*, then created an in vitro model of *E. coli* infection using murine monocytic RAW264.7 cells. In this study, a high number of macrophages were recruited to the colon of infected mice, as expected in an inflammatory process. Further research was conducted based on the crucial role of H_2_S in numerous pathophysiological conditions. In fact, bacterial infection can cause changes in H_2_S synthases, but infection was found to be a highly dynamic process. Hence, various enzymes may catalyze distinct stages of an infection, resulting in the production of H_2_S. In this study, gene and protein expression results indicated that CSE expression fluctuation was stronger compared to CBS and MPST in infected cells, suggesting that CSE is a major H_2_S producer in macrophages during *E. coli* infection, particularly in 5 h infection. Previous studies have demonstrated significant increases in both CBS and CSE expression with the same *Mycobacterium Tuberculosis* (Mtb) [19, 20], while *Staphylococcus aureus* [21], *Helicobacter pylori* [22], and *Pseudomonas aeruginosa* [23] can result in an increase in CSE expression.

In previous studies, the role of CSE in inflammation concerning H_2_S production has been explored. However, there is no consensus on the matter and findings largely depend on exogenous versus endogenous enzymatic H_2_S derivation. For example, Rahman et al. demonstrated that CSE promotes an excessive innate immune response, suppresses the adaptive immune response, and reduces circulating IL-1β, IL-6, TNF-α, and IFN-γ levels in response to *Mtb* infection [19]. However, host CSE derived H_2_S protects against *Pseudomonas aeruginosa* sepsis [23]. The contrasting outcomes highlight the intricate and adaptable nature of the cellular immune reaction. In our research, we observed that CSE distinctly augmented inflammatory responses in macrophages, potentially linked to elevated ROS production and macrophage polarization.

Given that mammalian lipid droplets possess a protein-mediated antimicrobial capability, which is heightened during poly-microbial sepsis and in response to LPS [24, 25], and it is noteworthy that H_2_S has the capacity to increase both the size and quantity of lipid droplets while simultaneously reducing lipolysis [26]. HIF-1α, as a transcription factor, plays an important role between metabolic activities and immune response [27]. Firstly, we observed a significant decrease in HIF-1α expression after inhibiting CSE during *E. coli* infection. We then focused on lipid metabolism and found that PAG reduced the accumulation of lipid droplets in RAW264.7 macrophages. Additionally, we examined the gene expressions of several other factors related to lipid metabolism and observed that PAG decreased the expression of lipogenesis genes (*Srebf1* and *Srebp2*) while increasing the expression of steatolysis gene *Acox1*. It is known that AMPK can be activated through both LKB1 and LKB1-independent pathways [28], and there are multiple mechanisms through which H_2_S may impact LKB1 [29, 30]. As ACC acts downstream of AMPK in fatty acid metabolism, AMPK can inhibit ACC activity through phosphorylation, thereby reducing fatty acid synthesis. Despite this, we found that the phosphorylation of ACC increased instead of decreased upon adding PAG before and after *E. coli* infection in present study, which suggests that PAG may have a direct impact on ACC. However, the exact mechanism requires further investigation.

We propose that AMPK may activate ULK1 through phosphorylation, thereby regulating cellular autophagy. Recent studies indicate that endogenously produced and/or exogenously administered H_2_S may exhibit two opposing effects on autophagy in various disease models [31, 32]. These effects could be attributed to factors such as concentration, time frame, and reaction time of H_2_S, as well as differences between disease stages or models. It is generally believed that autophagy is positively correlated with LC3 and negatively correlated with p62. The former was markedly decreased and the latter was increased following CSE overexpression alone, which indicates that autophagic degradation is inactive. However different manifestations were found after *E. coli* infection, which led to the upregulated expression of autophagy related proteins. It is well-known that autophagy is a double-edged sword, and the exact threshold at which protective autophagy becomes cytotoxic autophagy remains unknown. For example, Kong et al. discovered that excessive autophagy disorders promote inflammatory responses in the pancreas [33]. Our data demonstrate that inhibition of autophagy by CQ and AMPK inhibitor compound C also significantly reduces the inflammatory response. This effect is consistent with previous findings that treatment with CSE inhibitors produces similar results. However, it is important to note that autophagy not only eliminates unnecessary or dysfunctional parts of the cell but also releases amino acids and molecules that support the cell's metabolic functions. Cysteine, for example, can be used as a substrate for the enzymatic creation of H_2_S via CSE. Inhibiting autophagy can directly hinder the production of CSE/H_2_S, which can lead to the alleviation of inflammation. Therefore, future studies should be performed to validate these conclusions.

Finally, researches have demonstrated that H_2_S produced by the intestinal microbiota can have the same effect as endogenously-produced H_2_S. This is noteworthy because intestinal cells are also exposed to H_2_S from the luminal side in addition to the H_2_S produced internally [34]. The results of monitoring the level of macrophage polarization are interesting because they show that the use of slow-releasing H_2_S donor GYY4137 reduces polarization in macrophages. This finding is in line with a previous study which demonstrated that GYY4137 is effective in inhibiting NLRP3 inflammasome activity in macrophages [35]. Furthermore, GYY4137 reduces the secretion of inflammatory factors and limits the production of ROS in cardiomyocytes [36].

**Figure 8:**
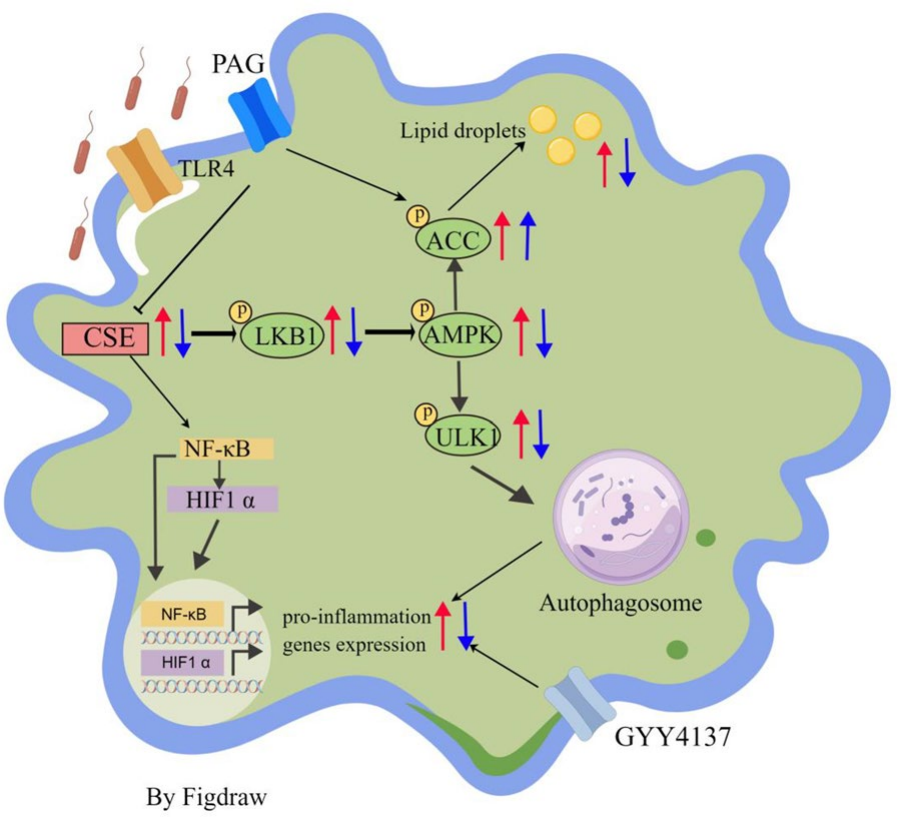
**Proposed mechanism for CSE/H**_**2**_**S exacerbating inflammation in macrophages.** Red arrows represent increase; blue arrows represent decrease.

In summary, our study highlights the significance of CSE in controlling the macrophage-mediated response to *E. coli* (Figure 8). Prolonged overexpression of CSE in macrophages results in inflammation upon exposure to *E. coli*. Alternatively, curbing CSE activity can curtail it, which could be connected to surplus autophagy. The use of PAG and GYY4137 could have crucial therapeutic applications for combating infections caused by *E. coli* and other related pathogens. Future studies could investigate the potential relationship between CSE and *Desulfovibrio*, which are known to be the major producers of exogenous H_2_S in the gastrointestinal tract. Additionally, it would be interesting to develop drugs that could regulate the generation of exogenous H_2_S.

### Supplementary Information

**Additional file 1**: **Key resources table**.

**Additional file 2**: **Oligonucleotide sequences used for qPCR.**

**Additional file 3**: **PAG reversed the observed effect on autophagy of CSE overexpression.** RAW264.7 cells were transfected with CSE overexpression plasmids, with or without PAG, while control cells were transfected with an empty vector. Immunoblots were performed to detect changes in autophagy-related proteins. Data are presented as the means ± SEMs (*n* = 3). *(*P* < 0.05) = significantly different.

## Data Availability

All data included in this study are available upon request by contact with the corresponding author.
