# Supplementary material for: The therapeutic potential for targeting CSE/H2S signaling in macrophages against Escherichia coli infection
Source: Vet Res. 2023 Aug 29;54:71. doi: 10.1186/s13567-023-01203-8 (PMC10466716; doi:10.1186/s13567-023-01203-8)
Supplement: Supplementary file 1 — Additional file 1: Key resources table. [file 13567_2023_1203_MOESM1_ESM.doc]

**Additional file 1 Key resources table**

| Reagent or Resource | Source | Identifier |
| --- | --- | --- |
| Antibodies | | |
| CSE | Proteinteck, Wuhan, China | Cat#12217-1-AP |
| CBS | Abclonal, Wuhan, China | Cat#A11612 |
| 3-MPST | Abclonal, Wuhan, China | Cat#A11587 |
| p-p65 | Abmart, Shanghai, China | Cat#TP56371F |
| P65 | Abmart, Shanghai, China | Cat#T55034F |
| iNOS | Abmart, Shanghai, China | Cat#[T55993](http://www.ab-mart.com.cn/page.aspx?node= 77 &id= 2228) |
| COX-2 | Sangon Bioteck, Shanghai, China | Cat#D323097 |
| P62 | Abcam, Cambridge, UK | Cat#ab109012 |
| Arg1 | Abcam, Cambridge, UK | Cat#ab133543 |
| HIF-1α | Abcam, Cambridge, UK | Cat#ab179483 |
| LC3B | Cell Signaling Technology, Boston, USA | Cat#3868S |
| β-tubulin (4D3) monoclonal antibody | Bioworld, Nanjing, China | Cat#AP0064 |
| Anti-rabbit IgG, HRP-linked antibody | Cell Signaling Technology, Boston, USA | Cat#7074S |
| Anti-mouse IgG, HRP-linked antibody | Cell Signaling Technology, Boston, USA | Cat#7076 |
| Chemicals | | |
| Difluoro{2-[1-(3,5-dimethyl-2H-pyrrole-2-ylidene-N)ethyl]-3,5-dimethyl-1H-pyrrolato-N}boron (BODIPY 493/503) | Sigma-Aldrich, MO, USA | Cat#790389CAS:121207-31-6 |
| GYY4137 | Sigma-Aldrich, MO, USA | Cat#SML0100CAS: 106740-09-4 |
| DL-Propargylglycine | Aladdin,Shanghai, China | Cat#[D133969](https://www.aladdin-e.com/zh_cn/d133969.html)CAS:64165-64-6 |
| Compound C | Selleck Chemicals, USA | Cat#S7306CAS:1219168-18-9 |
| [Chloroquine](https://www.tsbiochem.com/compound/chloroquine) | TargetMol, USA | Cat#T8689CAS:54-05-7 |
| Commercial Assays | | |
| Endogenous hydrogen sulfide (H_2_S) assay kit | Jiancheng, Nanjing, China | Cat#A146-1-1 |
| Total cholesterol (T-CHO) assay kit | Jiancheng, Nanjing, China | Cat#A111-1-1 |
| Triglyceride (TG) assay kit | Jiancheng, Nanjing, China | Cat#A110-1-1 |
| Lactate dehydrogenase (LDH) assay kit | Jiancheng, Nanjing, China | Cat#A020-2 |
| Experimental Models: Bacterial Strains | | |
| *Escherichia coli* | ATCC, Manassas, USA | Cat#ATCC 25922 |
| Experimental Models: Cell lines | | |
| Macrophage cell line (RAW264.7) | ATCC, Manassas, USA | Cat#TIB-71 |
| Software and Algorithms | | |
| GraphPad Prism v8 | GraphPad software | N/A |
| Image J v.2.0.0 | NIH | N/A |
| Other |  |  |
| DMEMB(1X) | Gibco, New York, USA | Cat#11965-065 |
| 0.25% trypsin-EDTA (1X) | Gibco, New York, USA | Cat#25200-056 |
| Fetal bovine serum | Gibco, New York, USA | Cat#10099-141 |
| 4,6-diamidino-2-phenylindole (DAPI) | Sigma-Aldrich, MO, USA | Cat#10236276001 |
| Polyvinylidene fluoride membrane | Millipore, Bedford, USA | N/A |
