# Supplementary material for: The therapeutic potential for targeting CSE/H2S signaling in macrophages against Escherichia coli infection
Source: Vet Res. 2023 Aug 29;54:71. doi: 10.1186/s13567-023-01203-8 (PMC10466716; doi:10.1186/s13567-023-01203-8)
Supplement: Supplementary file 2 — Additional file 2: Oligonucleotide sequences used for qPCR. [file 13567_2023_1203_MOESM2_ESM.docx]

**Additional file 2** **Oligonucleotide sequences used for qPCR.**

| **Gene** | **Primers sequence (5′-3′)** | **Orientation** |
| --- | --- | --- |
| ***Actin*** | TCTGGCACCACACCTTCTA | Forward |
|  | AGGCATACAGGGACAGCAC | Reverse |
| ***CSE*** | CAAAGCAACACCTCGCACTC | Forward |
|  | GCGGCTGTATTCAAAACCCG | Reverse |
| ***CBS*** | GAGGGGACTCAAGAGCCAAC | Forward |
|  | CCAGCAGAGCCATCTTCACA | Reverse |
| ***3-MPST*** | AGGAGCTGTGGTGAGGAGAT | Forward |
|  | AGGAGATGCTCAGATTGCGG | Reverse |
| ***HIF-α*** | ACCTTCATCGGAAACTCCAAA | Forward |
|  | CTGTTAGGCTGGGAAAAGTTA | Reverse |
| ***Il-1β*** | AACCTGCTGGTGTGTGACGTTC | Forward |
|  | CAGCACGAGGCTTTTTTGTTGT | Reverse |
| ***Il-6*** | CAAAGCCAGAGTCCTTCAGAG | Forward |
|  | GTCCTTAGCCACTCCTTCTG | Reverse |
| ***Tnf-α*** | CATCTTCTCAAAATTCGAGTGACAA | Forward |
|  | TGGGAGTAGACAAGGTAGAACCC | Reverse |
| ***Srebf1*** | GCGCTACCGGTCTTCTATCA | Forward |
|  | TGCTGCCAAAAGACAAGGG | Reverse |
| ***Srebf2*** | CCAAAGAAGGAGAGAGGCGG | Forward |
|  | CGCCAGACTTGTGCATCTTG | Reverse |
| ***Scd1*** | CCGAAGTCCACGCTCGAT | Forward |
|  | TGGAGATCTCTTGGAGCATGTG | Reverse |
| ***Fasn*** | GATCCTGGAACGAGAACAC | Forward |
|  | AGACTGTGGAACACGGTGGT | Reverse |
| ***Acox1*** | CGAGAAATCGAGAACTTG | Forward |
|  | CTTCGAGTGAGGAAGTTA | Reverse |
| ***Cd36*** | CCTGGGAGTTGGCGAGAAA | Forward |
|  | CGATCACAGCCCATTCTCCT | Reverse |
