# Supplementary figures and images for: The therapeutic potential for targeting CSE/H2S signaling in macrophages against Escherichia coli infection
Source: Vet Res. 2023 Aug 29;54:71. doi: 10.1186/s13567-023-01203-8 (PMC10466716; doi:10.1186/s13567-023-01203-8)

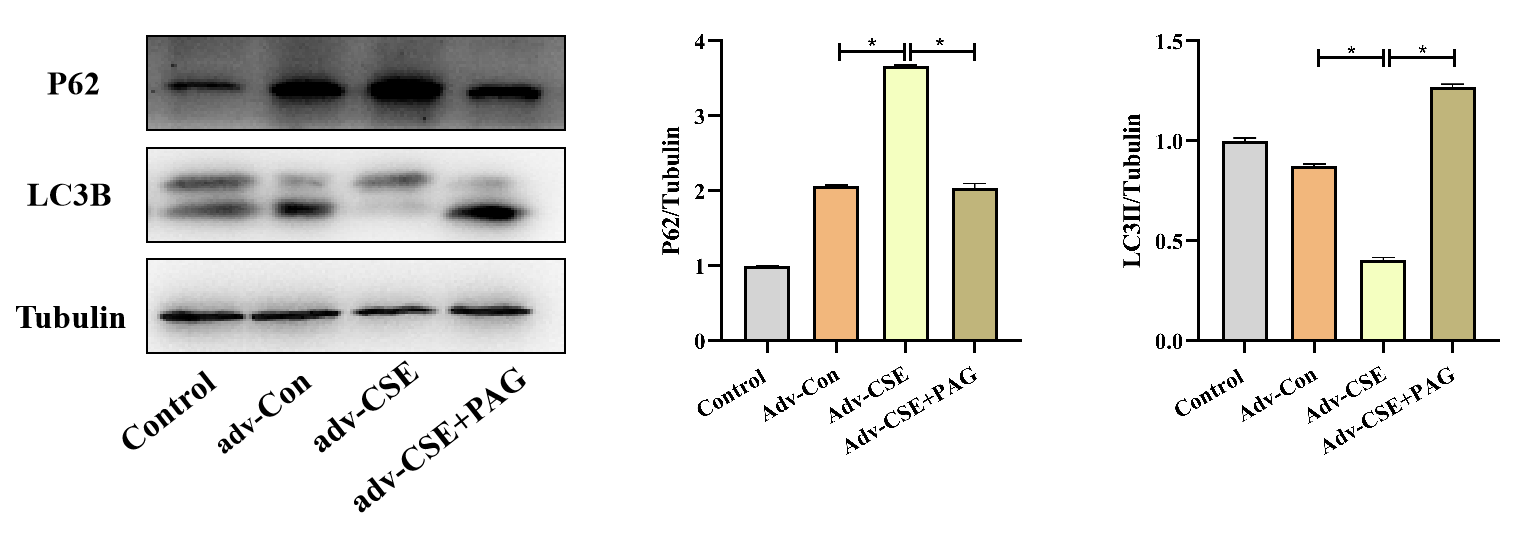

Supplement: Supplementary file 3 — Additional file 3: PAG reversed the observed effect on autophagy of CSE overexpression. RAW264.7 cells were transfected with CSE overexpression plasmids, with or without PAG, while control cells were transfected with an empty vector. Immunoblots were performed to detect changes in autophagy-related proteins. Data are presented as the means ± SEMs (n = 3). *(P < 0.05) = significantly different. [file 13567_2023_1203_MOESM3_ESM.docx]
